# Impact of additional counselling sessions through phone calls on smoking cessation outcomes among smokers in Penang State, Malaysia

**DOI:** 10.1186/1471-2458-14-460

**Published:** 2014-05-16

**Authors:** Ali Qais Blebil, Syed Azhar Syed Sulaiman, Mohamed Azmi Hassali, Juman Abdulelah Dujaili, Alfian Mohamed Zin

**Affiliations:** 1 Discipline of Clinical Pharmacy, School of Pharmaceutical Sciences, Universiti Sains Malaysia, 11800 Penang, Malaysia; 2 Department of Clinical Pharmacy, Faculty of Pharmaceutical Sciences, UCSI university, Jalan Menara Gading, UCSI Heights, 56000 Kuala Lumpur, Malaysia; 3 Discipline of Social and Administrative Pharmacy, School of Pharmaceutical Sciences, Universiti Sains Malaysia, 11800 Penang, Malaysia; 4 Quit Smoking Clinic, Penang General Hospital, Jalan Residensi, 10990 Penang, Malaysia

**Keywords:** Loading counselling, Smoking cessation, Malaysia

## Abstract

**Background:**

Studies all over the world reported that smoking relapses occur during the first two weeks after a quit date. The current study aimed to assess the impact of the additional phone calls counselling during the first month on the abstinence rate at 3 and 6 months after quit date among smokers in Penang, Malaysia.

**Methods:**

The study was conducted at Quit Smoking Clinic of two major hospitals in Penang, Malaysia. All the eligible smokers who attended the clinics between February 1^st^ and October 31^st^ 2012 were invited. Participants were randomly assigned by using urn design method either to receive the usual care that followed in the clinics (control) or the usual care procedure plus extra counselling sessions through phone calls during the first month of quit attempt (intervention).

**Results:**

Participants in our cohort smoked about 14 cigarettes per day on average (mean = 13.78 ± 7.0). At 3 months, control group was less likely to quit smoking compared to intervention group (36.9% vs. 46.7%, verified smoking status) but this did not reach statistical significance (OR = 0.669; 95% CI = 0.395-1.133, P = 0.86). However, at 6 months, 71.7% of the intervention group were successfully quit smoking (bio-chemically verified) compared to 48.6% of the control group (*P* < 0.001). The control group were significantly less likely to quit smoking (OR = 0.375; 95% CI = 0.217-0.645, *P* < 0.001).

**Conclusions:**

Smoking cessation intervention consisting of phone calls counselling delivered during the first month of quit attempt revealed significantly higher abstinence rates compared with a standard care approach. Therefore, the additional counselling in the first few weeks after stop smoking is a promising treatment strategy that should be evaluated further.

**Trial registration:**

TCTR20140504001

## Background

There is no specific method or technique that has been confirmed as the best way to improve the abstinence rates of smoking. Numerous studies have tried to find the most effective and feasible method to assist and support smokers who want to quit smoking. In Malaysia, the majority (80.3%) of tobacco users tried to stop smoking without assistance. Furthermore, 9% of them used pharmacotherapy followed by counselling (4.4%) or quit-line and others (7.6%) (Such as traditional medicines, switching to smokeless tobacco) [1].

Several studies reported that up to 70% of the tobacco users who quit smoking cigarettes had relapsed within the first two weeks of their quit dates [2-4]. Thus, the initial two weeks are considered a window period prior to the achievement of long-term abstinence. After this critical era, curves of relapse of the treatment and control groups in intervention studies become parallel [5-7]. Thus, the prevention of relapse in the first two weeks might enhance the probability of successful long-term abstinence [8].

The majority of smokers prefer to cease using tobacco products, avoiding attendance at counselling sessions or group programmes (person-to-person contact) [9-11]. Counselling through phone calls has been paid more attention as an alternative for the delivery of services [12-15]. There are numerous potential benefits of smoking cessation telephone counselling including the ease of use, whenever required and wherever the tobacco user is located; cost-effective delivery and scalability to a large number of people, regardless of location; the ability to tailor messages to key user characteristics (such as age, sex, ethnicity); providing a content that can distract the smoker from craving; and linking the smokers with others for social support [16].

Therefore, in order to provide a more comfortable and feasible intervention and to assess additional counselling sessions during the first month after the quit date, it has been interesting to evaluate the effectiveness of this type of counselling using telephone calls. The results will help to explore and assess the benefits of using loading counselling sessions using telephone calls to support people who want to quit, and to compare this method with the usual provisions in Malaysian health care settings regarding the abstinence rate.

## Methods

### Setting and participants

The study was carried out at the Quit Smoking Clinic of two major hospitals in Penang state, Pulau Pinang and Seberang Jaya Hospitals. All eligible cigarettes smokers who attended the clinics between February 1^st^ and October 31^st^ of 2012 were invited.

### Inclusion and exclusion criteria

All individuals who attended the clinics during the period under review were invited to participate in the research. Inclusion criteria involved: (1) New registered smokers who attend the Quit Smoking Clinic (either walk-in, referred from the outpatient clinics from the same hospital, or referred from outside primary clinics), (2) Male or female aged ≥ 18 years old, (3) Willing to stop smoking. In addition, all outpatient clinics of both hospitals were contacted to refer any smoker patient willing to stop smoking to Quit Smoking Clinic.

While the exclusion criteria consisted of: (1) Any inpatient referred to the Quit Smoking Clinics, (2) Recent (three months or below) history of serious cardiac arrhythmia, angina pectoris, myocardial infarction, or other medical conditions that from researchers' view participants might not be commitment with the study, (3) Currently using NRT or other smoking cessation treatments (bupropion or varenicline) within the last 12 months before study enrolment, (4) Use tobacco products other than cigarettes, (5) Pregnant, lactation or intend to be pregnant, (6) Use of Psychoactive drugs, (7) Suspected drug or alcohol abuse, (8) Patients who continue to buy nicotine gum after the first 2 weeks of treatment (as a normal policy in these hospitals, the patients are supplied free nicotine gum for 2 weeks only after that if the patients wants to continue use NRT must buy it themselves).

### Study design

**Figure 1:**
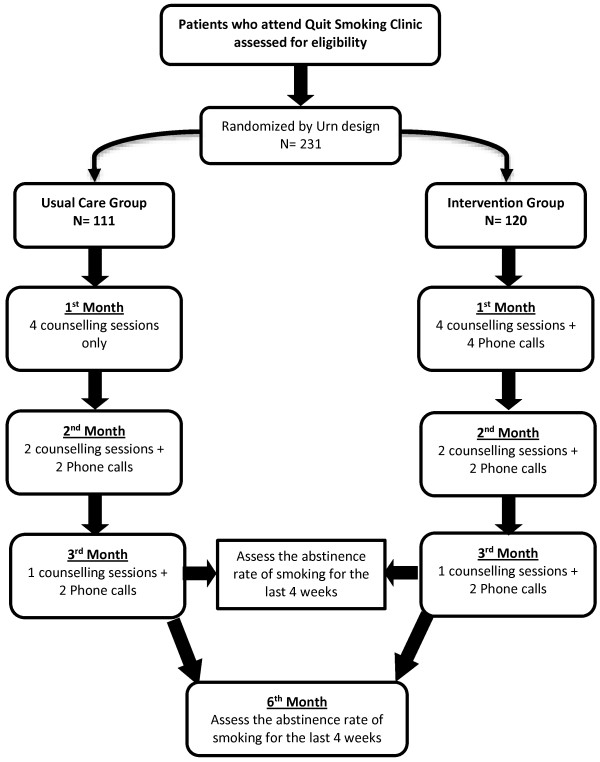
Schematic presentation of the study design.

Participants were randomly assigned using urn design to receive either the usual care (a combination of nicotine gum and cognitive behaviour therapy); (control group), or the previous combination plus extra counselling sessions through phone calls during the first month of the quit attempt (intervention group) (Figure 1). The purpose and required adherence to the study protocol were explained to the participants, and signed consent was obtained from each participant by expert counsellors. The expert counsellors at quit smoking clinic of both hospitals who were specialists in delivering smoking cessation services and who were in charged to provide counselling support as a part of the national health services accepted to cooperate in the present study.

### Sample size

A minimum sample size of 123 subjects per group was powered to detect a difference of at least 11% between the intervention group and control group. Allowing for 20% dropout, the final sample size was 148 subjects per arm. This was based on data from a study by Ron Borland *et al.*, which compared the effectiveness of a telephone call-back counselling intervention with the provision of self-help resources alone for quitting smoking [17]. The total target sample size was 296 subjects.

### Randomisation

The selection of the method to randomise the patients to treatment groups is one of the important statistical issues in comparative trials. Although the target sample size of the current trial was determined beforehand, the actual number of participants recruited into the trial may not be known in advance. Urn design was used for randomisation because it have the ability to impose a small trial to be balanced but acts like complete randomisation as the size of the trial increases. Consequently, the assignments of treatment within a sequence created by the urn design are not as predictable as those of other types of restricted randomisation processes, and the susceptibility to bias is likewise reduced [18].

### Procedure

After the assessment for the eligibility, the participants were randomised and allocated to the specific groups. All participants during the baseline visit were interviewed by the expert counsellor. The counsellor interviewed them using intense counselling approach for about 20–30 minutes. The study participants were asked about their socio-demographic (age, sex, race, marital status, education level), medical background, and smoking-related information history (age started to smoke, number of cigarettes smoked per day, previous quit attempt), the level of nicotine dependence using the Malay version of Fagerstrom Test for Nicotine Dependence (FTND-M) [19] and their confidence in quitting by self-reported efficacy (i.e. how they rate their chance of stopping smoking permanently at the current attempt) using a 5-point Likert scale (very high, high, neutral, low and very low), and exhaled carbon-monoxide (CO) level (higher intensity counselling refers to an intervention that involves extended contact between the patient and the clinician of more than 10 minutes [20,21]). The exhaled CO level was measured by using the Smokerlyzer MicroCO® meter (Micro Medical Limited Company). The concentration of CO from the breath was measured, with a cut-off point CO level of ≥ 7 part per million (ppm) as indicative of smoking.

The smokers learnt about the negative effects of smoking and their effect on health as well as the positive effect from stopping it. In addition, all participants received free nicotine gum supplements for 2 weeks only according to general regulations and policies followed in the Ministry of Health Malaysia. They learnt how to use NRT, gained knowledge about smoking withdrawal symptoms and how to control themselves against any of these withdrawal symptoms or cravings for cigarette smoking, especially during the first two weeks after quitting. Moreover, each smoker received self-help materials in the form of brochures and booklets on stopping smoking in Malay and Chinese languages to help him/her review what he/she has learnt from the counsellor. These materials have been developed and published by the Health Education Division, Ministry of Health Malaysia, and reported the harmful effects and health consequences of smoking. In addition, one of the booklets displays the objectives of the Quit Smoking Clinic, including clinic processes to achieve its goals and other activities.

The participants, who randomised to the control group, received the usual care which is recommended by the Ministry of Health, Malaysia. According to Malaysian clinical practice guidelines for the treatment of tobacco use and dependence the counsellor conduct individual counselling for the smoker who wants to quit by providing him all the support and advices as well as self-help materials [22] (see Figure 1). During the counselling session, which last for 20–30 minutes, the counsellor emphasised and reviewed problem solving and training procedures for each participants to support quitting [22].

For those in the usual care group, during the first month of the study, the participants attended the Quit Smoking Clinic four times with a gap of one week between each visit. In the second month, the participants were asked to attend the clinic once every two weeks (totally 2 visits per month) and received an extra phone call one week after each visit (totally 2 phone calls per month). Furthermore, during the third month, participants received two phone calls and were asked to attend the clinic at the end of the month.

In the intervention group, the smokers received the same care procedure, except that they received an extra proactive phone call each week besides attending the clinic only in the first month (i.e., four visits + extra four phone calls). The procedure for the other two months was the same as for the control group.

During the extra phone call counselling sessions, the counsellor encouraged smokers to endure with their attempt to quit and concentrated on their success so far. He provided them information about the harmful effects of smoking and provided them positive feedback and emphasised the benefits of stop smoking. He encouraged the participants to cease the usage of cigarettes, lighters, and ashtrays, and to avoid any environment where they would usually smoke. Furthermore, the counsellor prompted the smokers to identify the obstacles and the difficulties of stop smoking and strategy how to overcome them. The counsellor talked for about 10–15 minutes with every participant in each proactive extra phone call.

The main objective for the study was determining the self-reported point-prevalence abstinence (the previous 4 weeks) verified by exhaled CO level at 3 and 6 months follow-up points. At the end of the third month, the tobacco users were asked about their smoking status and were asked to attend the Quit Smoking Clinic again after 3 months (6 months from the baseline visit) to assess smoking status (4 weeks-point prevalence abstinence). Participants who chose not to attend the visit at the end of the third month or the visit at the end of the sixth month were considered smokers at that point (at the end of three month, 7 participants in control group and 3 participants in intervention were lost to follow-up. While at the 6-month follow-up point, 13 in the control group and 5 in the intervention group were lost). The outcomes data was collected by another research member not connected with counselling and the data analysis.

### Statistical analysis

The collected data was analysed using SPSS version 18.0 (SPSS Inc., Chicago, IL). The level of significance was set at *P* value less than 0.05. Descriptive statistics were used to display the baseline characteristics for the whole sample population, including the socio-demographic and smoking-related information. The continuous variables were shown as mean ± standard deviation, while the frequencies and proportions were used in order to describe the categorical variables. The comparability of demographic and smoking-related information between the control group and the intervention group was assessed using independent-sample *t*-test or Mann–Whitney U test were used for continuous variables, and Pearson’s chi-square (χ^2^) and Fisher’s exact test for categorical variables.

Smoking cessation outcomes were compared between the intervention group and the control group using Mann–Whitney U test and Fisher’s exact test at third and sixth months. These nonparametric statistical tests were also performed to compare smoking abstinence rates (4 weeks point prevalence abstinence) between the two groups at 3- and 6-month follow-up points. In addition, binary logistic regression was employed to predict the impact of the extra phone calls (the integrated intervention) on the smoking cessation outcomes. The effectiveness of additional counselling in the first month of the quit attempt compared with the usual care on the prevalence of smoking abstinence was assessed through odds ratio (OR), and binary logistic regression was used to control the effect of the confounder.

### Ethical approval

The present study was approved by the Institute of Public Health (IPH), the National Institutes of Health (NIH) and the Medical Research and Ethics Committee (MREC) of the Ministry of Health, Malaysia.

## Results

### The baseline characteristics of the study participants

Corresponding to 283 subjects identified between February 1^st^ and October 31^st^ 2012, 231 smokers accepted to participate in the study. Unfortunately, recruitment procedure was prematurely stopped because of time and financial restriction as well as the involvement of the Clinics in a large project organised by the Ministry of Health, Malaysia.

Of 231 participants, 111 (48.1%) smokers were randomly assigned to the usual care group, while the rest (120 smokers) of them allocated to the intervention group. The mean age with standard deviation (SD) of the participants at the time of enrolment into the study was 48.26 ± 13.7 years (range 18 – 77 years) (Table 1). The majority of our cohort was Chinese (114/231). The vast majority of the study participants were male (96.1%) and 67.5% were married. In addition, 67.5% of our subjects were referred from the outpatient clinics of the same hospital. Furthermore, majority of the study participants had a low level of education (more than 90% finished either primary or secondary school only).

**Table 1:** Demographic and smoking-related characteristics of tobacco users (N = 231) in quit smoking clinics, Penang, Malaysia

**Characteristics**	**Value**
***Participants’ category***	
Referred from outside-hospital clinic	70 (30.3%)
Referred from outpatient clinic	156 (67.5%)
Walk-in	5 (2.2%)
***Age*** (mean ± SD) (years)	48.26 ± 13.7
***Weight*** (mean ± SD) (kg)	62.09 ± 8.4
***Sex***	
Female	9 (3.9%)
Male	222 (96.1%)
***Race***	
Malay	88 (38.1%)
Chinese	114 (49.4%)
Indian	29 (12.5%)
***Marital Status***	
Single	42 (18.2%)
Divorced/Widow	33 (14.3%)
Married	156 (67.5%)
***Education status***	
Primary	109 (47.1%)
Secondary	100 (43.2%)
Collage/University	21 (9.1%)
No formal education	1 (0.6%)
***Smoking habits/history***	
Age started smoking (mean ± SD)	17.38 ± 3.9
Duration of smoking (mean ± SD)	31.03 ± 13.7
No. of Cigarettes smoked/day (mean ± SD)	13.78 ± 7.0
**Breath Co level** (ppm) (mean ± SD)	14.45 ± 4.9
**FTND-M score** (mean ± SD)	3.79 ± 1.2
**Previous smoking attempt**	
No	203 (87.9%)
Yes	28 (12.1%)
**Other smokers in household**	
No	150 (64.9%)
Yes	81 (35.1%)

*Note*. SD = Standard Deviation; CO = carbon monoxide; ppm = part per million; FTND = Fagerström test for nicotine dependence.

Two hundred and twenty one (95.7%) of the study participants had a history of chronic diseases such as hypertension, diabetes mellitus, asthma, and heart disease. The highest frequency among these comorbidities was hypertension (98 patients) then diabetes mellitus and dyslipidaemia (66 patients for each).

The mean age of starting smoking in the study population was 17.38 ± 3.9 years (range 8 – 35 years). The tobacco users smoked about 14 cigarettes per day on average (mean = 13.78 ± 7.0; range 3 – 50 cigarettes per day). In addition, the mean FTND-M score was 3.79 ± 1.2 which considers low addiction according to the score range of FTND-M. The majority of the study participants did not try to quit smoking in the past (87.9%).

Moreover, there were no significant differences between the control and intervention groups with regards to baseline socio-demographics and smoking history data (Table 2). However, group type (control versus intervention group) was significantly associated with the participants’ category (*P* = 0.025), i.e. walk-in, referred from the outpatient clinics from the same hospital, or referred from outside primary clinics, as the majority of the subjects in both group were referred from out-patient clinics of the same hospital. Thus, this baseline difference between groups was controlled in subsequent analyses.

**Table 2:** Demographic and smoking-related characteristics of control versus intervention groups

**Characteristics**	**Group type**		***P *** **value**^**a**^
	**Control**	**Intervention**	
	**N = 111**	**N = 120**	
***Participants’ category***			0.025
Referred from outside-hospital clinic	40 (36.0%)	30 (25.0%)	
Referred from outpatient clinic	71 (64.0%)	85 (70.8%)	
Walk-in	0 (0%)	5 (4.2%)	
***Age*** (mean ± SD) (years)	48.30 ± 13.6	48.22 ± 13.8	0.964^b^
***Weight*** (mean ± SD) (kg)	62.77 ± 6.6	61.46 ± 9.8	0.499^c^
***Sex***			0.502^d^
Female	3 (2.7%)	6 (5.0%)	
Male	108 (97.3%)	114 (95.0%)	
***Race***			0.063
Malay	49 (44.1%)	39 (32.5%)	
Chinese	53 (47.7%)	61 (50.8%)	
Indian	9 (8.1%)	20 (16.7%)	
***Marital Status***			0.086
Single	22 (19.8%)	20 (16.7%)	
Divorced/Widow	10 (9.0%)	23 (19.2%)	
Married	79 (71.2%)	77 (64.2%)	
***Education status***			0.718
Primary	54 (48.6%)	55 (45.8%)	
Secondary	46 (41.4%)	54 (45.0%)	
Collage/University	11 (9.9%)	10 (8.3%)	
No formal education	0 (0%)	1 (0.8%)	
***Chronic Disease***			0.519
No	6 (5.4%)	4 (3.3%)	
Yes	105 (94.6%)	116 (96.7%)	
***Smoking habits/history***			
No. of Cigarettes smoked/day	14.54 ± 7.6	13.08 ± 6.3	0.111^c^
Age started smoking	17.52 ± 4.07	17.24 ± 3.8	0.931^c^
Duration of smoking	31.09 ± 13.5	30.97 ± 13.9	0.946^b^
***Breath Co level***	14.93 ± 5.2	14.00 ± 4.4	0.300^c^
***FTND-M score***	3.88 ± 1.2	3.71 ± .1	0.323^c^
**Other smokers in household**			0.214^d^
No	77 (69.4%)	73 (60.8%)	
Yes	34 (30.6%)	47 (39.2%)	
***Previous smoking attempt***			0.105^d^
No	102 (91.9%)	101 (84.2%)	
Yes	9 (8.1%)	19 (15.8%)	
***Chance rate to quit***			0.916
Very high	0 (0%)	0 (0%)	
High	28 (25.2%)	33 (27.5%)	
Neutral	67 (60.4%)	71 (59.2%)	
Low	16 (14.4%)	16 (13.3%)	
Very low	0 (0%)	0 (0%)	

^a^Chi-square (χ^2^) test, ^b^Independent sample *t*-test, ^c^Mann–Whitney U test, ^d^Fisher’s exact test.

### Smoking cessation interventions and outcome monitoring

Participants were monitored during the follow-up visits in the third and sixth months for smoking status (4-week point prevalence abstinence), exhaled CO level as a marker to verify quitting, and number of cigarettes smoked per day in case the participant was still smoking.

The smoking cessation outcomes were compared between the study groups and are presented in Table 3. At the three months follow-up visit after the quitting date, participants who received the additional counselling in the first moth had significantly higher rate of success in quitting smoking when compared with those who received the usual care alone depending on self-reporting the 4-week point prevalence abstinence (57.5% vs. 44.1%, respectively: *P* = 0.049). However, the smoking status for the participants after verification of exhaled CO level did not significantly differ between the study groups.

**Table 3:** Smoking cessation outcomes of the control versus intervention conditions

**Outcomes**	**Group type**		***P *** **value**^**a**^
	**Control**	**Intervention**	
	**N = 111**	**N = 120**	
***Third month monitoring***			
**Self-reported 4-week point prevalence abstinence**			0.049
Yes	49 (44.1%)	69 (57.5%)	
No	62 (55.9%)	51 (42.5%)	
**Breath CO level**	7.75 ± 3.9	7.24 ± 3.6	0.334^b^
**Smoking status (verified)**			0.144
Non smoker	41 (36.9%)	56 (46.7%)	
Smoker	70 (63.1%)	64 (53.3%)	
***Sixth month monitoring***			
**Self-reported 4-week point prevalence abstinence**			< 0.001
Yes	57 (51.4%)	89 (74.2%)	
No	54 (48.6%)	31 (25.8%)	
**Breath CO level**	7.12 ± 4.3	5.73 ± 3.5	0.005^b^
**Smoking status (verified)**			< 0.001
Non smoker	54 (48.6%)	86 (71.7%)	
Smoker	57 (51.4%)	34 (28.3%)	

^a^Fisher’s exact test; ^b^Mann–Whitney U test.

In addition, 6 months after the quit date, the self-reported 4-week point prevalence was significantly higher in the intervention group compared with those in the control group (74.2% vs. 51.4%, respectively: *P* < 0.001). Similarly, the verified smoking status was significantly different between the usual care and combination of usual care and extra phone calls (48.6% vs. 71.7%, respectively: *<* 0.001).

### Risk estimation of integrated intervention on the smoking cessation outcomes

At the three month follow-up point, the participants in the usual care group were less likely to quit smoking, but this did not reach statistical significance (OR = 0.669; 95% CI = 0.395-1.133, *P* = 0.86) (Table 4). In addition, the intervention cohort group was 1.209 times more likely to quit smoking when compared to the control group (95% CI = 0.945-1.545). Similarly, at six months, the control group was 0.375 times less likely to quit smoking (95% CI = 0.217-0.645, *P* < 0.001).

**Table 4:** Risk estimation of quit smoking with regards to group type at 3 and 6 months follow-up points

**Outcomes**	**Group type**		**OR**	**95% CI**	***P *** **value**
	**Control**	**Intervention**			
	**N = 111**	**N = 120**			
***At 3 months***					
**Smoking status**			0.669^a^	0.395-1.133	0.86
Non smoker	41 (36.9%)	56 (46.7%)			
Smoker	70 (63.1%)	64 (53.3%)			
**Smoking status**			0.732^b^	0.420-1.512	0.26
Non smoker	41 (36.9%)	56 (46.7%)			
Smoker	70 (63.1%)	64 (53.3%)			
***At 6 months***					
**Smoking status**			0.375^a^	0.217-0.645	< 0.001
Non smoker	54 (48.6%)	86 (71.7%)			
Smoker	57 (51.4%)	34 (28.3%)			
**Smoking status**			0.376^b^	0.224-0.671	0.001
Non smoker	54 (48.6%)	86 (71.7%)			
Smoker	57 (51.4%)	34 (28.3%)			

*Note*. OR = odds ratio; CI = confidence interval.

^a^Crude odds ratio, ^b^Adjusted odds ratio for participants category.

After controlling for the effect of confounder (participants’ category) by using binary logistic regression, the usual care group was less likely to report non-smoking at the 3 and 6 months follow-up points. Although, at 3 months the result failed to reach statistical significance (adjusted OR = 0.732, *P* = 0.255).

## Discussion

### Demographic characteristics of the study participants

The tobacco users of the current study were predominantly male (96.1%). This probably reflects the previously reported statistics of the NHMS III. Cigarette smoking is tremendously rare among Malaysian females [23]. Furthermore, the report of the Global Adult Tobacco Survey (GATS) in Malaysia reported that 22.9% of individuals aged 15 years or older (43.6% of males and 1% of females) were current smokers of cigarettes including manufactured, hand-rolled, or kreteks [1]. Numerous studies reported that while cigarettes smoking remains acceptable for males, smoking by women is not socially sanctioned in Malaysia and other Asian countries in general [24-26]. This suggests that quitting for female smokers is unlikely because they did not reveal the truth about their smoking status; hence the study was dominated by men.

The majority of the study participants had a history chronic disease (such as hypertension and diabetes mellitus). This supports the general thought that the majority of tobacco users intended to stop smoking when they encountered medical health problems. In 2011, GATS Malaysia reported that about one-third of the population of current smokers and former smokers who had been abstinent for less than one year had visited a health care provider. Out of them, 68% had been asked by their health care providers if they smoked and about 53% had been recommended to stop smoking by their clinicians [1]. Therefore, more attention and extra effort should be focused on increasing the health awareness program about smoking and risk-associated with smoking even for healthy individual.

The majority of our participants were referred by either outpatient clinics or outside clinics. Surprisingly, no tuberculosis patients were referred to the Quit Smoking Clinics during the data collection of the current study. Although, local studies reported that smoking prevalence among tuberculosis patients was high and ranged from 40.3% to 53.4% in the state of Penang, Malaysia [27,28]. Furthermore, smoking is an independent predictor of poor tuberculosis treatment outcomes and prognosis [28] and that integrating tobacco cessation intervention in tuberculosis care confer advantages on the outcomes of tuberculosis treatment [29]. Therefore, more attention should be given to this group of patient.

### Effect of the integrated intervention on smoking cessation outcomes

Finding from the current study demonstrated that self-reported point prevalence abstinence in the intervention arm was significantly higher than those in the control arm at 3 and 6 months. However, a biochemically-verified abstinence rate among the participants experiencing loading counselling through phone calls was higher in the subjects of the intervention group (74.2% versus 51.4%) and this was statistically significance at the end of 6 months only after the quit date (*P* < 0.001). Despite the believed objectivity of the CO biochemical measure, it does not provide a gold standard nor is it a perfect of accuracy in assessing smoking status [30]. Since smoking by-products have short half-life in the body, the biochemical assessment of smoking status using Smokerlyzer is verified for the time only near the specimen collection (i.e., 24–48 hours) [31]. However, some participants didn’t pass the biochemical assessment at the end of 3 and 6 months and therefore were considered smoker. This suggests that smoking validation using CO as a chemical biomarker resulted in the evaluation and the conformation of the self-report accuracy.

In addition, the subjects in the intervention group were more likely to be abstinent (using verified 4-week point prevalence abstinence) at the end of three and six months. However, it was statistically significant at the end 6 months (OR = 1.644, 95% CI = 1.222 - 2.212). Our finding suggesting that more intensive counselling during the critical period of quitting attempt (*i.e.* during the first month) could have a beneficial impact on long term. Similarly, a study was conducted in the United States, where people living with HIV/AIDS were randomised either to usual smoking cessation treatment or to a cell phone intervention. In the intervention group, the phone calls spanned a period of 3 months but were front loaded such that the majority of the phone calls were near the time of the scheduled quit date. At the end of third months, the patients in the intervention group were 4.3 (95% CI = 1.9 - 9.8) times more likely to quit smoking (7-day point prevalence abstinence) compared with the usual care participants [32]. Another study that compared the efficacy of front loaded versus weekly behavioural smoking cessation used a counselling schedule to increase the likelihood of successful early abstinence and subsequent long-term abstinence among 278 adult tobacco users [33]. At the end of 12 months post-quitting, intervention participants were more likely to be abstinent when compared with the participants in the weekly counselling schedule with regard to continuous abstinence, 7 or more consecutive days nor for 7 or more consecutive episodes, and point prevalence abstinence. However, it was significant only when continuous abstinence was used (11.7% vs. 6.3%, *P* = 0.007) [33].

Another study reported that the strength of motivation to quit smoking, but not dependence, predicted success at abstinence [34]. This raises the possibility that in countries such as Malaysia that are at an earlier stage in the “tobacco epidemic” [35] motivation plays a vital role in success at quitting than dependence. This could happen if there are still large numbers of tobacco users who find it relatively easy to quit.

A potential limitation of the current study is the relatively small sample size (the supposed target sample size 296 subjects, while the collected sample size was 231 subjects). However, by using the urn design randomisation, this problem was resolved to ensure randomisation between groups. Another limitation is that 96% of the recruited participants were males. Therefore, the generalizability of our results to their counterpart females might be compromised. Applying a single biomarker for the verification of the smoking status might result in misclassification and using an analytical method involved two or more biomarker (cotinine in plasma, saliva, or urine; thiocyanate in plasma or saliva; and CO in expired air) to distinguish objectively between smokers and non-smokers is recommended [36]. However, collection of sample involves more contact with participants than usual in field studies may result in increased refusal [37]. Due to this and financial restriction, only exhaled CO level was used for the biochemical assessment. Moreover, researches demonstrated that spouses impact each other behaviours. Since the smoking rate in Malaysian women is low and the majority of our cohort was male, this may affect the decision to quit smoking of their male spouse. Other external factors may influence the subject’s decision to quit smoking such as the increasing cost of living, price of goods including cigarettes, and others. These factors might contribute to the difference observed in the abstinent rate between the 3^rd^ and 6^th^ month follow up period, however we cannot fully understand the effect of these factors from the current data.

## Conclusion

In summary, Smoking cessation intervention consisting of extra sessions of phone call counselling during the first month of a quit attempt results in significantly higher abstinence rates compared with a standard care approach. While the results from the current study are encouraging, 12-month outcome analyses will be needed to better evaluate the effectiveness of this kind of loading approach at long term. Therefore, this study provides evidence that additional counselling during the first few weeks after the quit date is a promising treatment strategy that should be evaluated further.

## Competing interests

The authors declare that they have no competing interests.

## Authors’ contributions

AQB contributed to the design of the study and wrote the first draft, AMZ contributed to the collection of outcome measure data, AQB and JAD contributed to data analysis and reviewed manuscript, SASS and MAH reviewed the manuscript, SASS supervised the writing and contributed to the discussion. All authors read and approved the final manuscript.

## Pre-publication history

The pre-publication history for this paper can be accessed here:

http://www.biomedcentral.com/1471-2458/14/460/prepub

## References

[B1] Institute for Public Health Report of the Global Adult Tobacco Survey (GATS) Malaysia, 2011 Ministry of Health Malaysia 2012

[B2] Garvey AJ Kinnunen T Nordstrom BL Utman CH Doherty K Rosner B Vokonas PS Effects of nicotine gum dose by level of nicotine dependence Nicotine Tob Res 2000 2 1 53 63 10.1080/14622200050011303 11072441

[B3] Hughes JR Keely J Naud S Shape of the relapse curve and long-term abstinence among untreated smokers Addiction 2004 99 1 29 38 10.1111/j.1360-0443.2004.00540.x 14678060

[B4] Jorenby DE Smith SS Fiore MC Hurt RD Offord KP Croghan IT Hays JT Lewis SF Baker TB Varying nicotine patch dose and type of smoking cessation counseling JAMA 1995 274 17 1347 1352 10.1001/jama.1995.03530170027027 7563558

[B5] Shiffman S Ferguson S Gwaltney C Immediate hedonic response to smoking lapses: relationship to smoking relapse, and effects of nicotine replacement therapy Psychopharmacology (Berl) 2006 184 3–4 608 618 16283258 10.1007/s00213-005-0175-4

[B6] Hays JT Hurt RD Rigotti NA Niaura R Gonzales D Durcan MJ Sachs DPL Wolter TD Buist AS Johnston JA White JD Sustained-release bupropion for pharmacologic relapse prevention after smoking cessation a randomized, controlled trial Ann Intern Med 2001 135 6 423 433 10.7326/0003-4819-135-6-200109180-00011 11560455

[B7] Yasin SM Moy FM Retneswari M Isahak M Koh D Timing and risk factors associated with relapse among smokers attempting to quit in Malaysia Int J Tuberc Lung Dis 2012 16 7 980 985 10.5588/ijtld.11.0748 22507850

[B8] Zhu SH Stretch V Balabanis M Rosbrook B Sadler G Pierce JP Telephone counseling for smoking cessation: effects of single-session and multiple-session interventions J Consult Clin Psychol 1996 64 1 202 211 8907100 10.1037//0022-006x.64.1.202

[B9] Fiore MC Novotny TE Pierce JP Giovino GA Hatziandreu EJ Newcomb PA Surawicz TS Davis RM Methods used to quit smoking in the United States. Do cessation programs help? JAMA 1990 263 20 2760 2765 10.1001/jama.1990.03440200064024 2271019

[B10] McGoven PG Lando HA Roski J Pirie PL Sprafka JM A comparison of smoking cessation clinic participants with smokers in the general population Tob Control 1994 3 4 329 333 10.1136/tc.3.4.329

[B11] Stead LF Lancaster T Behavioural interventions as adjuncts to pharmacotherapy for smoking cessation Cochrane Database of Systematic Reviews 2012: John Wiley & Sons, Ltd 10.1002/14651858.CD009670.pub2 23235680

[B12] Anderson DM Duffy K Hallett CD Marcus A Cancer prevention counseling on telephone helplines Public Health Rep 1992 107 3 278 283 1594737 PMC1403647

[B13] DeBusk RF Miller NH Superko HR Dennis CA Thomas RJ Lew HT Berger WE Heller RS Rompf J Gee D A case-management system for coronary risk factor modification after acute myocardial infarction Ann Intern Med 1994 120 9 721 729 10.7326/0003-4819-120-9-199405010-00001 8147544

[B14] Lando HA Hellerstedt WL Pirie PL McGovern PG Brief supportive telephone outreach as a recruitment and intervention strategy for smoking cessation Am J Public Health 1992 82 1 41 46 10.2105/AJPH.82.1.41 1536333 PMC1694411

[B15] Orleans CT Schoenbach VJ Wagner EH Quade D Salmon MA Pearson DC Fiedler J Porter CQ Kaplan BH Self-help quit smoking interventions: effects of self-help materials, social support instructions, and telephone counseling J Consult Clin Psychol 1991 59 3 439 448 2071729 10.1037//0022-006x.59.3.439

[B16] Whittaker R McRobbie H Bullen C Borland R Rodgers A Gu Y Mobile phone-based interventions for smoking cessation Cochrane Database of Systematic Reviews 2012: John Wiley & Sons, Ltd 10.1002/14651858.CD006611.pub3 23152238

[B17] Borland R Segan CJ Livingston PM Owen N The effectiveness of callback counselling for smoking cessation: a randomized trial Addiction 2001 96 6 881 889 10.1046/j.1360-0443.2001.9668819.x 11399219

[B18] Wei LJ Lachin JM Properties of the urn randomization in clinical trials Control Clin Trials 1988 9 4 345 364 10.1016/0197-2456(88)90048-7 3203525

[B19] Anne YHA Ng CG Rusdi AN Validation of the Malay version of Fagerstrom test for nicotine dependence (FTND-M) among a group of male staffs in a University Hospital Malays J Psychiatry 2011 20 1

[B20] Fiore MC Jaén CR Baker TB Bailey WC Benowitz NL Curry SJ Dorfman SF Froelicher ES Goldstein MG Healton CG Treating Tobacco Use and Dependence: 2008 Update 2008 5 Rockville (MD): U.S. Department of Health and Human Services, Public Health Service, Agency for Healthcare Research and Quality

[B21] Casagrande J Pike M Smith P An improved approximate formula for calculating sample sizes for comparing two binomial distributions Biometrics 1978 34 3 483 486 10.2307/2530613 719125

[B22] Disease Control Division Clinical Practice Guideline on Treatment of Tobacco Use and Dependence 2003 Ministry of Health Malaysia 2003

[B23] Ministry of Health Malaysia Institute of Public Health Malaysia Report of the Third National Health and Morbidity Survey (NHMS III) 2006

[B24] Morrow M Barraclough S Tobacco control and gender in south-east Asia. Part II: Singapore and Vietnam Health Promot Int 2003 18 4 373 380 10.1093/heapro/dag403 14695368

[B25] Morrow M Barraclough S Tobacco control and gender in Southeast Asia. Part I: Malaysia and the Philippines Health Promot Int 2003 18 3 255 264 10.1093/heapro/dag021 12920146

[B26] Tsai Y-W Tsai T-I Yang C-L Kuo KN Gender differences in smoking behaviors in an Asian population J Womens Health 2008 17 6 971 978 10.1089/jwh.2007.0621 PMC2942753 18681817

[B27] Awaisu A Nik Mohamed M Abd Aziz N Syed Sulaiman S Mohamad Noordin N Muttalif A Ahmad Mahayiddin A Tobacco use prevalence, knowledge, and attitudes among newly diagnosed tuberculosis patients in Penang State and Wilayah Persekutuan Kuala Lumpur, Malaysia Tob Induc Dis 2010 8 1 1 10 10.1186/1617-9625-8-1 20148105 PMC2819235

[B28] Dujaili JA Sulaiman SAS Awaisu A Muttalif A Blebil AQ Outcomes of tuberculosis treatment: a retrospective cohort analysis of smoking versus non-smoking patients in Penang, Malaysia J Public Health 2011 19 2 183 189 10.1007/s10389-010-0365-3

[B29] Awaisu A Nik Mohamed MH Mohamad Noordin N Abd Aziz N Syed Sulaiman SA Muttalif AR Ahmad Mahayiddin A The SCIDOTS project: evidence of benefits of an integrated tobacco cessation intervention in tuberculosis care on treatment outcomes Subst Abuse Treat Prev Policy 2011 6 1 26 10.1186/1747-597X-6-26 21943384 PMC3196696

[B30] Patrick DL Cheadle A Thompson DC Diehr P Koepsell T Kinne S The validity of self-reported smoking: a review and meta-analysis Am J Public Health 1994 84 7 1086 1093 10.2105/AJPH.84.7.1086 8017530 PMC1614767

[B31] Benowitz NL Grubowski J, Bell CS The use of biologic fluid samples in assessing tobacco smoke consumption Measurement in the Analysis and Treatment of Smoking Behavior. Volume NIDA research monograph 48 1983 Washington, DC: US: Department of Health and Human Services 6 26 6443145

[B32] Vidrine DJ Marks RM Arduino RC Gritz ER Efficacy of cell phone–delivered smoking cessation counseling for persons living with HIV/AIDS: 3-month outcomes Nicotine Tob Res 2012 14 1 106 110 10.1093/ntr/ntr121 21669958 PMC3242970

[B33] Garvey AJ Kalman D Hoskinson RA Kinnunen T Wadler BM Thomson CC Rosner B Front-loaded versus weekly counseling for treatment of tobacco addiction Nicotine Tob Res 2012 14 5 578 585 10.1093/ntr/ntr256 22058190 PMC3337538

[B34] Wee LH West R Bulgiba A Shahab L Predictors of 3-month abstinence in smokers attending stop-smoking clinics in Malaysia Nicotine Tob Res 2011 13 2 151 156 10.1093/ntr/ntq221 21186253

[B35] Lopez AD Collishaw NE Piha T A descriptive model of the cigarette epidemic in developed countries Tob Control 1994 3 3 242 10.1136/tc.3.3.242

[B36] Torano JS van Kan HJM Simultaneous determination of the tobacco smoke uptake parameters nicotine, cotinine and thiocyanate in urine, saliva and hair, using gas chromatography–mass spectrometry for characterisation of smoking status of recently exposed subjects Analyst 2003 128 7 838 843 10.1039/b304051h 12894819

[B37] Velicer WF Prochaska JO Rossi JS Snow MG Assessing outcome in smoking cessation studies Psychol Bull 1992 111 1 23 41 1539088 10.1037/0033-2909.111.1.23

